# Nϵ-Carboxymethyl-Lysine Deteriorates Vascular Calcification in Diabetic Atherosclerosis Induced by Vascular Smooth Muscle Cell-Derived Foam Cells

**DOI:** 10.3389/fphar.2020.00626

**Published:** 2020-05-15

**Authors:** Sui-Ning Xu, Xin Zhou, Cun-Jun Zhu, Wei Qin, Jie Zhu, Ke-Lin Zhang, Hui-Jin Li, Lu Xing, Kun Lian, Cheng-Xiang Li, Zhen Sun, Zhong-Qun Wang, An-Ji Zhang, Hui-Ling Cao

**Affiliations:** ^1^Department of Cardiology, The First Affiliated Hospital, Shaanxi Key Laboratory of Ischemic Cardiovascular Disease, Institute of Basic and Translational Medicine, Xi’an Medical University, Xi’an, China; ^2^Department of Cardiology, Xijing Hospital, The Fourth Military Medical University, Xi’an, China; ^3^Department of Cardiology, Affiliated Luan Hospital of Anhui Medical University, Luan, China; ^4^Department of Cardiology, Affiliated Hospital of Jiangsu University, Zhenjiang, China

**Keywords:** Nϵ-carboxymethyl-lysine (CML), vascular calcification (VC), diabetic atherosclerosis, vascular smooth muscle cell (VSMC), foam cell

## Abstract

Nϵ-carboxymethyl-lysine (CML), an advanced glycation end product, is involved in vascular calcification (VC) in diabetic atherosclerosis. This study aimed to investigate the effects of CML on VC in diabetic atherosclerosis induced by vascular smooth muscle cell (VSMC)–derived foam cells. Human studies, animal studies and cell studies were performed. The human study results from 100 patients revealed a poor blood glucose and lipid status and more severe coronary lesions and stenosis in patients with coronary artery disease and diabetes mellitus. Intraperitoneal injection of streptozotocin combined with a high-fat diet was used to build a diabetic atherosclerosis model in ApoE^−/−^ mice. The animal study results indicated that CML accelerated VC progression in diabetic atherosclerosis by accelerating the accumulation of VSMC-derived foam cells in ApoE^−/−^ mice. The cell study results illustrated that CML induced VSMC-derived foam cells apoptosis and aggravated foam cells calcification. Consistent with this finding, calcium content and the expression levels of alkaline phosphatase, bone morphogenetic protein 2 and runt-related transcription factor 2 were significantly elevated in A7r5 cells treated with oxidation-low-density lipoprotein and CML. Thus, we concluded that CML promoted VSMC-derived foam cells calcification to aggravate VC in diabetic atherosclerosis, providing evidence for the contribution of foam cells to diabetic VC.

## Introduction

Approximately 387 million people suffered from diabetes mellitus (DM) in 2014 according to the International Diabetes Federation, and this number is predicted to increase to 552 million by 2030 (Yamagishi et al., 2015). Additionally, vascular calcification (VC) is the main risk factor for adverse cardiovascular events and contributes to a high morbidity in DM patients (Benjamin et al., 2017; Stabley and Towler, 2017; Yahagi et al., 2017; Lino et al., 2018). VC refers to the phenotypic transdifferentiation of osteo-/chondroblasts from vascular smooth muscle cells (VSMCs) under pathological conditions including atherosclerosis, DM, and chronic kidney disease (CKD), through matrix vesicle secretion and hydroxyapatite crystals formation (Wang et al., 2020). Therefore, VC have a similar composition to bone minerals. VC can be classified into two distinct forms according to the location: intimal calcification and medial calcification. The link between DM and VC is driven by small muscular peripheral arteries (Ho and Shanahan, 2016). Medial calcification is the most common form of VC in DM and is associated with oxidative stress, active endothelial proinflammatory and proosteogenic conditions, such as physiological dysfunction, alterations in mineral metabolism, increased inflammatory cytokine production, and the release of osteoprogenitor cells from the marrow into the circulation (Yahagi et al., 2017).

Advanced glycation end products (AGEs) are the most important metabolites of diabetic glucose toxicity and participate in multiple stages of diabetic cardiovascular complications (Wang et al., 2014; Ye et al., 2016). Nϵ-carboxymethyl-lysine (CML) is considered the key component of AGEs. Previous studies have demonstrated that CML can be regarded as an early indicator of the diabetic VC (Xu et al., 2017; Li et al., 2017). Furthermore, our research group successfully built animal and cell models of diabetic VC to simulate the appearance of focal microcalcifications in the anterior tibial artery of patients with diabetic foot amputation (Wang et al., 2012; Wang et al., 2016).

The formation and accumulation of foam cells serve as important processes in atherosclerosis (AS). The formation of macrophage-derived foam cells in the intima is a major hallmark of early-stage AS lesions. Analogous to original foam cells, non-macrophage-derived foam cells engulf and process modified lipoproteins in lipid-rich conditions. Moreover, VSMCs have been reported to contribute more than 50% of foam cell populations in human AS lesions (Allahverdian et al., 2014). Dedifferentiated VSMCs migrating into the subintimal space of AS lesions could provide structural integrity for fibrous cap. Meanwhile, VSMCs can perform the function of cholesterol uptake in the lipid core (Liu et al., 2017). Considerable progress has been achieved in diabetic VC induced by macrophage-derived foam cells, but little research has been conducted on VC induced by VSMC-derived foam cells. Here, our study explores the effects of CML on VC in diabetic AS induced by VSMC-derived foam cells and provides evidence for the contribution of foam cells to diabetic VC.

## Materials and Methods

### Materials

A7r5 VSMCs were purchased from the Shanghai Cell Bank of the Chinese Academy of Sciences (Shanghai, China). Dulbecco’s modified Eagle’s Medium, Nutrient Mixture F-12 (DMEM/F12) was obtained from Gibco (Grand Island, USA). CML was acquired from Polypeptide Laboratories (San Diego, USA). Streptozotocin (STZ) was obtained from Sigma–Aldrich Co. LLC (St. Louis, USA). A CML ELISA kit was obtained from Meixuan Biological Science and Technology Co., Ltd (Shanghai, China). A calcium assay kit and alkaline phosphatase (ALP) activity kit were purchased from Nanjing Jiancheng Bioengineering Institute (Nanjing, China). A Von Kossa staining kit was purchased from Shunbai Biologicals Inc. (Shanghai, China). An Annexin V-FITC apoptosis detection kit was obtained from Sigma-Aldrich Co. LLC (St. Louis, USA). Glyceraldehyde-phosphate dehydrogenase (GAPDH) was acquired from Cell Signaling Technology, Inc. (Boston, USA). Antibodies against ALP, bone morphogenetic protein 2 (BMP-2) and runt-related transcription factor 2 (Runx2) and all secondary antibodies were from Santa Cruz Biotechnology (Santa Cruz, USA).

### Human Studies

The study was approved by the Ethical Committee of the First Affiliated Hospital of Xi’an Medical University and carried out in accordance with the institutional guidelines. Written informed consents were obtained from all patients. The inclusion criteria were established according to the 2013 European Society of Cardiology (ESC) guidelines (Montalescot et al., 2013). Patients with stable angina or other symptoms associated with coronary artery disease (CAD) diagnosed by coronary angiography (CAG). The exclusion criteria included patients with acute coronary syndrome, organic valvular disease, cardiac arrest, severe neurological disease, tumor, pregnancy, perioperative haemodynamic instability and a lack of the laboratory biochemical indicators needed for this study. Then, one hundred patients with CAD in the Department of Cardiology, the First Affiliated Hospital of Xi’an Medical University (Xi’an, China), were recruited from January to August 2019. The patients were classified into two groups according to whether they had a history of DM. The patients were consecutively recruited to the CAD group or CAD with DM group, and each group consisted of fifty patients. The DM patients had been diagnosed in accordance with the 1999 World Health Organization (WHO) diagnostic criteria (Alberti and Zimmet, 1998), and all enrolled DM patients were receiving antiglycaemic therapy (17 patients received insulin therapy and 33 patients received oral hypoglycaemic treatment).

The risk factors of the CAD patients and their profiles were evaluated, including age, sex, hypertension history, smoking status. And their fasting glucose (FPG), glycated hemoglobin (HbA1c), low-density lipoprotein cholesterol (LDL-C), high-density lipoprotein cholesterol (HDL-C), total cholesterol (TC), and triglyceride (TG) were measured with well-established methods (Duque et al., 2017). The serum CML concentration was measured by the CML ELISA kit according to the manufacturer’s instruction.

CAG was performed according to standard procedures. The angiograms were reviewed by at least two experienced cardiologists who reached an agreement on the disease origin and course according to the Science Clubs of America (SCA). Patients with significant stenosis of luminal narrowing ≥ 50% were considered as CAD. Significant AS was defined as luminal narrowing ≥75% detected in a main branch of the epicardial coronary arteries. The patients in this study were categorized as having significant single-, double-, or triple-vessel disease when a significant lesion of one or more coronary artery branches was found on CAG. The major coronary arteries were defined as the right coronary artery (RCA), left circumflex artery (LCX), and left anterior descending (LAD) coronary artery. We selected the LAD as the typical site of coronary artery lesions.

### Animal Studies

The animal study protocols in the study were approved by the Institutional Animal Care and Use Committee of Jiangsu University (Jiangsu, China) and conducted in accordance with the Guidelines for Animal Experimentation of the National Institutes of Health. ApoE^−/−^ mice with a C57BL/6J background were purchased from Jackson Laboratory (USA). All the mice were housed at 25°C under 12-h light and dark cycles and provided with regular chow. At 6 weeks of age, the mice were used to build a diabetic model through intraperitoneal injection of 40 mg/kg STZ for five consecutive days. After 2 weeks, the mice with blood glucose levels ≥16.7 mmol/L were defined as DM. These mice were then switched to a semisynthetic high-fat diet (HFD) (21% fat, 0.15% cholesterol, and other components were similar to those of regular chow) and injected with CML (10 mg/kg/day) through the tail vein once every other week for two months. A total of 30 ApoE^−/−^ mice were divided into three groups (10 mice per group): the control group (normal saline, regular chow), the STZ group (STZ, HFD) and the STZ + CML group (STZ+CML, HFD). After 4 months, all the mice were euthanized for serology analysis (serum glucose and serum CML levels) and morphological analysis [hematoxylin-eosin (H&E) staining, Von Kossa staining, and immunohistochemical staining]. Quantitative analyses of indicated stains were performed using Image J software.

### Histology and Immunohistochemistry

Aorta tissue was fixed using 10% formalin, dehydrated and embedded in paraffin. Some sections were stained with H&E. Images were taken under a light microscope and analyzed by investigators blinded to the treatment conditions. Calcium deposition in atherosclerotic plaques and VSMC calcification were identified by Von Kossa staining. First, VSMCs and the paraffin-embedded sections were fixed in 4% paraformaldehyde for 15 min at room temperature and washed with double distilled water (ddH_2_O) twice, followed incubated with 5% and 2% silver nitrate solution. Next, the specimens were placed in the dark under ultraviolet light for 30 min at room temperature. After removing the silver nitrate solution and washing with ddH_2_O twice, 5% sodium thiosulfate was used to remove the unreacted silver for 5 min, and the specimens were finally counterstained with eosin (tissue) or neutral red (cell) for 10 min and washed twice with ddH_2_O. Images were obtained with an Olympus microscope (IX51, Olympus, Japan).

For immunohistochemistry, the methods were performed as follows: first, aorta tissues were dewaxed and hydrated, and 3% H_2_O_2_ was used to inactivate endogenous peroxidases for 10 min. Then, protein-blocking agent was incubated with the slides for 10 min, followed by application of the primary antibody for incubation overnight at 4°C. Next, the sections were incubated with poly-horseradish peroxidase (HRP)-conjugated secondary antibody for 1 h at 37°C, and peroxidase activity was identified by a reaction with 3,3-diaminobenzidine tetra hydrochloride for 7 min. Finally, the sections were counterstained with hematoxylin.

### Cell Culture and Calcification Induction

A7r5 cells were divided into three groups: the control group (DMEM-F12), the ox-LDL group (DMEM-F12+ 50 μg/ml ox-LDL) and the ox-LDL+CML group (DMEM-F12+ 50 μg/ml ox-LDL+ 10 μmol/L CML). Calcification of VSMC-derived foam cells was induced as follows: first, the A7r5 cells were cultured until confluent and treated with ox-LDL or ox-LDL and CML for 24 h. Then, the cells were incubated with a calcification medium (DMEM-F12 with 10% fetal bovine serum, 10 mmol/L sodium pyruvate, 10^-7^ mol/L insulin, 10^-8^ mol/L dexamethasone, 100 U/ml penicillin, 100 mg/ml STZ, and 10 mmol/L β-glycerophosphate (β-GP) for two weeks in a humidified atmosphere with 5% CO_2_ at 37°C. During this period, the medium was replaced with fresh calcification medium every 48 h to 72 h.

### Oil Red O Staining

Cell culture was the same as above. After rinsing with phosphate-buffered solution (PBS) and 60% isopropanol for 5 min, VSMCs were fixed in 4% paraformaldehyde for 30 min at room temperature. Then, fresh-filtered oil red O working solution was used to stain the samples for 30 min at room temperature, and the staining was subsequently evaluated under an inverted microscope (IX51, Olympus, Japan).

### Measurement of Cellular Cholesterol Contents

The free cholesterol (FC) and TC contents of the collected cells were quantified by a modified enzymatic fluorometric method. Lipid extracts were dissolved in isopropanol and incubated with an enzyme mixture for 1 h (FC) or 2 h (TC) at 37°C, and then 0.1 M NaOH was added for 30 min. Fluorescence intensity was measured at excitation and emission wavelengths of 320 nm and 407 nm, respectively. FC and TC values were obtained from the standard calibration curves. The amount of cholesterol ester (CE) was calculated by subtracting FC from TC.

### Apoptotic Cell Detection by Flow Cytometry

The Annexin V-FITC apoptosis detection kit (Sigma-Aldrich, St. Louis, USA) was used to detect apoptosis according to the instructions of the manufacturer. The process was completed in the dark. First, A7r5 cells were cultured with ox-LDL or ox-LDL and CML for 24 h, and then the cells were collected into glass tubes. Next, the cells were stained with 10 μl of Annexin V solution at 37°C for 15 min, followed by staining with 10 μl of PI at 37°C for 15 min. Finally, the cells were quantified using flow cytometry (FACSCanto II, Becton Dickinson, USA).

### ALP Activity Assay and Quantification of the Calcium Content

First, the total proteins of cells were extracted by centrifugation in radioimmunoprecipitation assay (RIPA) lysis buffer (0.2% NP-40 in l mM MgCl_2_), and then the ALP activity was evaluated using an ALP assay kit based on the manufacturer’s instruction. Determined by the Bradford method, the results for ALP activity were normalized to the level of the total protein (Wei et al., 2020).

The calcium content was determined by colorimetry assays as previously described (Yahagi et al., 2017). After rinsing with PBS twice, dried aorta tissues or cells were decalcified with 0.6 M HCl for 24 h at room temperature. The calcium content was determined by a reaction with o-Cresolphthalein complexon (QuantiChrom TM Calcium Assay Kit, Bio Assay Systems, USA). Next, the samples were washed three times with PBS, and then dissolved in 0.1 M NaOH and 0.1% sodium dodecyl sulfate (SDS). The protein content was measured using the Bradford method. Finally, the calcium content of the samples was normalized to the total protein content.

### Western Blotting

Total proteins of A7r5 cells treated with ox-LDL and β-GP or ox-LDL, CML, and β-GP were extracted by centrifugation in RIPA lysis buffer. The protein samples were loaded onto SDS-polyacrylamide gel and then transferred onto polyvinylidene difluoride (PVDF) membranes using a semidry method. The membranes were incubated with the primary antibodies anti-BMP-2, anti-Runx2, anti-ALP, and anti-GAPDH overnight at 4°C and then with HRP-conjugated secondary antibodies for 1 h at room temperature. The immunoreactive bands were visualized with an ECL kit (Thermo Fisher Scientific, Rockford, USA), and images were obtained using Gel-Pro Analyzer 4 software (Media Cybernetics, USA).

### Statistical Analyses

All data were presented as the mean ± standard deviation (SD). Differences for multiple groups were compared by one-way ANOVA analysis followed by *post hoc* individual comparisons. An unpaired Student’s t test was applied to determine differences between two variables. *P* < 0.05 was considered as statistically significant. All statistical analyses were carried out using GraphPad 5.01 software (GraphPad Software Inc., USA).

## Results

### Baseline Clinical Data and Coronary Angiography in the CAD Patients

The one hundred recruited CAD patients were classified into two groups according to whether they had DM. Each group consisted of 50 patients. All the 50 DM patients were receiving antiglycaemic therapy. The patients’ baseline characteristics are listed in **Table 1**. Significant differences in the baseline characteristics LDL-C, FPG, HbA1c, and CML were identified between the two groups, but no significant differences in TC, TG, and HDL-C were found. Hypertension history and smoking status did not differ between the two groups.

**Table 1:** Baseline clinical data of CAD patients or combined with DM.

Groups	CAD Group	CAD+DM Group	*P* value
**Variables**	50	50	
**Diabetes (%)**	0(0)	50(1)	<0.0001
**Gender,M/(M+F)**	37/50	36/50	1
**Age(y)**	62.58 ± 0.81	66.65 ± 0.35	0.012
**Hypertension(%)**	27(54)	30(60)	0.686
**Smoke status(%)**	23(46)	22(44)	1
**TC(mmol/L)**	3.775 ± 0.133	3.47 ± 0.113	0.083
**TG(mmol/L)**	1.545 ± 0.109	1.726 ± 0.129	0.288
**HDL-C(mmol/L)**	0.9652 ± 0.028	0.9372 ± 0.03	0.497
**LDL-C(mmol/L)**	2.354 ± 0.118	1.972 ± 0.093	0.012
**FPG (mmol/L)**	5.316 ± 0.192	7.73 ± 0.348	0.001
**HbA1c (%)**	5.2 ± 0.3	7.4 ± 0.4	0.005
**CML(ng/ml)**	25.46 ± 2.99	28.72 ± 3.77	0.0125

Values are expressed as the number (%) or means ± SD. n = 50 for each group. Annotations: CAD, coronary artery disease; DM, diabetic mellitus; LDL-C, low-density lipoprotein cholesterol; HDL-C, high-density lipoprotein cholesterol; TC, triglyceride; TG, total cholesterol; FPG, fasting glucose; HbA1c, glycated hemoglobin; CML, Nϵ-carboxymethyl-lysine.

**Figure 1:**
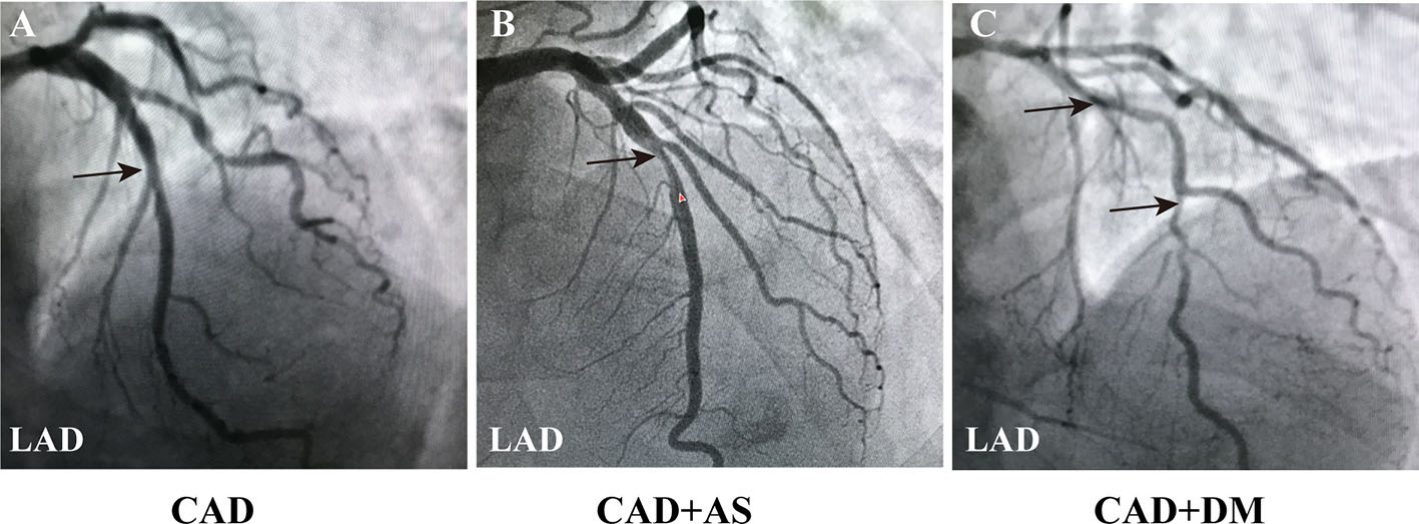
Coronary angiography images in CAD patients. **(A)** Angiography image of a CAD patient (LAD: 50%, LCX: 30%, RCA: 25%). **(B)** Angiography image of a CAD patient with a significant AS of single coronary vascular lesions (LAD: 75%, LCX: 30%, RCA: 25%). **(C)** Angiography image of a CAD patient combined DM with a serious atherosclerosis of three coronary vascular lesions (LAD: 90%, LCX: 70%, RCA: 80%-90%). The results indicated more severe coronary lesions and stenosis in patients with CAD and DM. Black arrowheads indicate LAD coronary artery stenosis. Annotations: CAD, coronary artery disease; DM, diabetic mellitus; LAD, left anterior descending coronary artery; LCX, left circumflex coronary artery; RCA, right coronary artery; AS, atherosclerosis.

The CAG results of the CAD patients are shown in **Figure 1**. Luminal narrowing of 50% in the middle section of the LAD is shown in **Figure 1A**, which was indicative of CAD. Luminal narrowing of 75% in the middle section of the LAD and 30% in an area near LAD are shown in **Figure 1B**, which were defined as significant AS and served as an indication for coronary stent implantation in the CAD patient. Furthermore, luminal narrowing of 90% in the middle section of the LAD, 70% in the middle section of the LCX and 80%–90% in the middle section of RCA are shown in **Figure 1C**, which reflected serious atherosclerosis of three coronary vascular lesions in a CAD patient with DM.

### Progression of Atherosclerotic Calcification in Diabetic ApoE^−/−^ Mice

**Figure 2:**
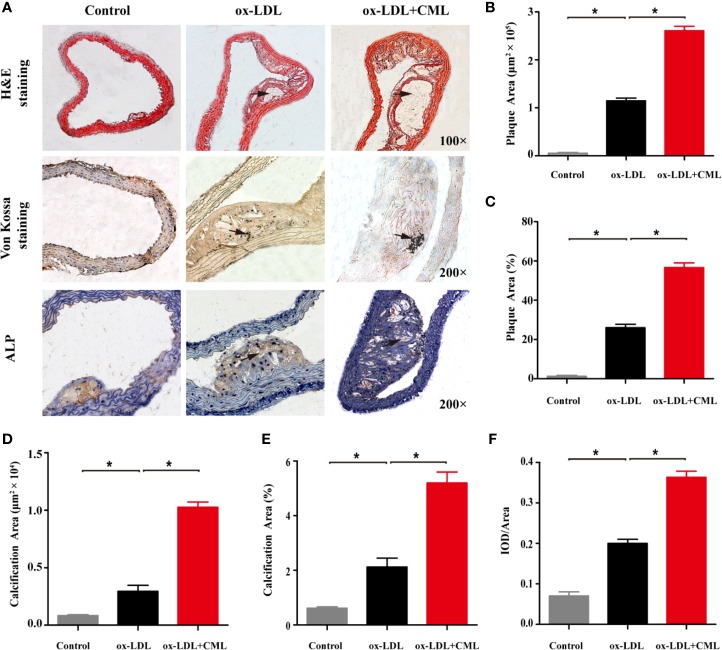
CML exacerbated atherosclerotic calcification in diabetic ApoE^−/−^ mice. **(A)** Representative photomicrographs of atherosclerotic lesions in aortic cross-sections after H&E staining (black arrowheads indicate atherosclerotic lesions, 100× magnification), Von Kossa staining (black arrowheads indicate black calcium particles, 200× magnification) and immunohistochemical staining for ALP (black arrowheads indicate brown positive staining areas, 200× magnification). Quantitative results of H&E staining for plaque area **(B)** and the ratio of plaque area **(C)**. Quantitative results of Von Kossa staining for calcification area **(D)** and the ratio of calcification area **(E)**. Quantitative results of immunohistochemical staining for ALP **(F)**. Values are expressed as the mean ± SD. *P < 0.05 .Annotation: CML, Nϵ-carboxymethyl-lysine; ALP, alkaline phosphatase. IOD, integrated optical density.

The morphological characteristics of aortic AS plaques in ApoE^−/−^ mice were visualized by H&E staining (**Figures 2A–C**). The aortic intima of the control group was locally thickened, and early AS plaques were observed in the ox-LDL group, but no destruction of the internal elastic plate was observed. The area of aortic plaque in the ox-LDL+CML group was significantly larger than that in the ox-LDL group and a large number of cholesterol crystals were visible under the fibrous cap. Von Kossa staining was performed to describe VC (**Figures 2A, D, E**). A small number of black calcium particles were observed in the ox-LDL group, while extensive spotty calcium deposits were noted in the ox-LDL+CML group, which indicating that CML aggravated VC.

ALP plays an important role in VC, and upregulated expression of ALP indicates severe VC. We used immunohistochemical staining to observe ALP expression in AS plaques, and brown sediments indicated positive staining. Almost no ALP expression was observed in the control group. A small amount of brown staining corresponding to ALP expression was found in the arterial plaques of the ox-LDL group. ALP expression in the arterial plaques of the ox-LDL+CML group was significantly increased, implying that CML exacerbated VC (**Figures 2A, F**).

### CML Levels in ApoE^-/-^ Mice

Changes in the serological indications of the ApoE^−/−^ mice were found. Serum CML levels gradually rose in the control group, ox-LDL group and the ox-LDL+CML group (9.51 ± 0.87 vs. 14.56 ± 1.53 vs. 20.81 ± 3.22, *P* < 0.05, n=10). Likewise, serum Glu levels exhibited the similar trend in the control group, ox-LDL group and the ox-LDL+CML group (12.24 ± 1.69 vs. 21.67 ± 1.23 vs. 25.56 ± 2.26, *P* < 0.05, n=10).

### CML Promoted the Formation of A7r5 VSMC-Derived Foam Cells

**Figure 3:**
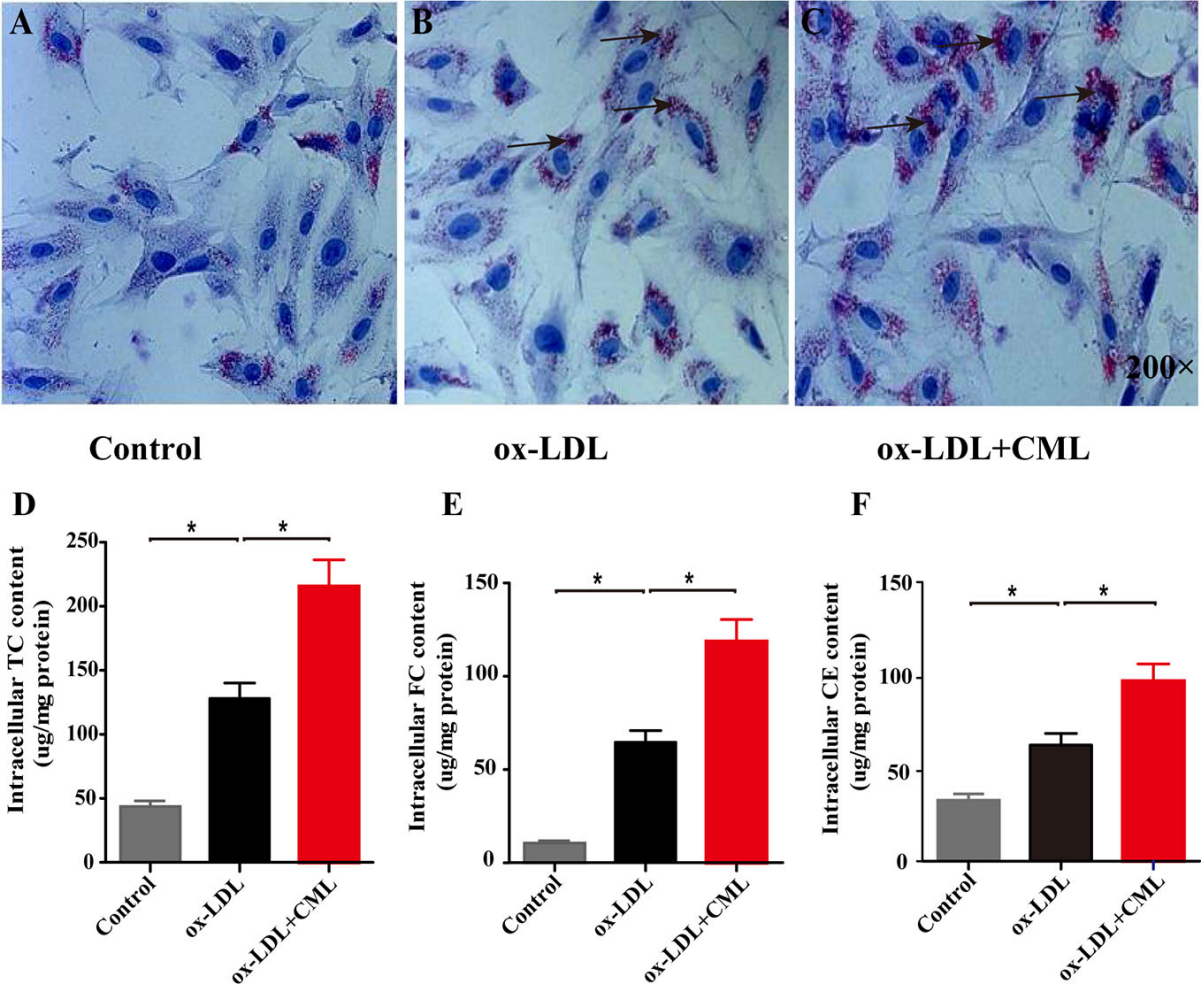
CML accelerated the lipid accumulation of A7r5 VSMC-derived foam cells. The degree of lipid accumulation was evaluated by oil red O staining (black arrowheads indicate lipid accumulation, 200× magnification). **(A)** Control group; **(B)** ox-LDL group; **(C)** ox-LDL+CML group. Intracellular cholesterol content by enzymatic method for **(D)** TC, **(E)** FC and **(F)** CE. Values are expressed as the mean ± SD. *P < 0.05. Annotations: CML, Nϵ-carboxymethyl-lysine; VSMC, vascular smooth muscle cell; ox-LDL, oxidation-low density lipoprotein; TC, total cholesterol; FC, free cholesterol; CE, cholesterol ester.

A7r5 VSMC-derived foam cells were formed by VSMCs loaded with ox-LDL or ox-LDL and CML. Oil red O staining was performed to observe lipid accumulation. Few lipid droplets were observed in the control group as shown by oil red O staining (**Figure 3A**), demonstrating a low intracellular lipid content in the control group. A number of red lipid droplets in the cytoplasm were found in the ox-LDL group, which indicated the formation of foam cells (**Figure 3B**). The red lipid droplets in the ox-LDL+CML group were significantly increased compared with those in the ox-LDL group (**Figure 3C**). Consistent with the oil red O staining, the amounts of cholesterol gradually increased in the control group, ox-LDL group, and ox-LDL+CML group, namely, TC (43.56% ± 4.42% vs. 128.10% ± 12.06% vs. 215.57% ± 20.69%), FC (10.73% ± 1.09% vs. 64.63% ± 6.31% vs. 118.70% ± 11.70%), and CE (32.83% ± 3.35% vs. 62.43% ± 6.31% vs. 96.87% ± 9.17%), respectively (**Figures 3D–F**; *P* < 0.05), which disclosed that CML aggravated the formation of A7r5 VSMC-derived foam cells.

### CML Augmented the Apoptosis of A7r5 VSMC-Derived Foam Cells

**Figure 4:**
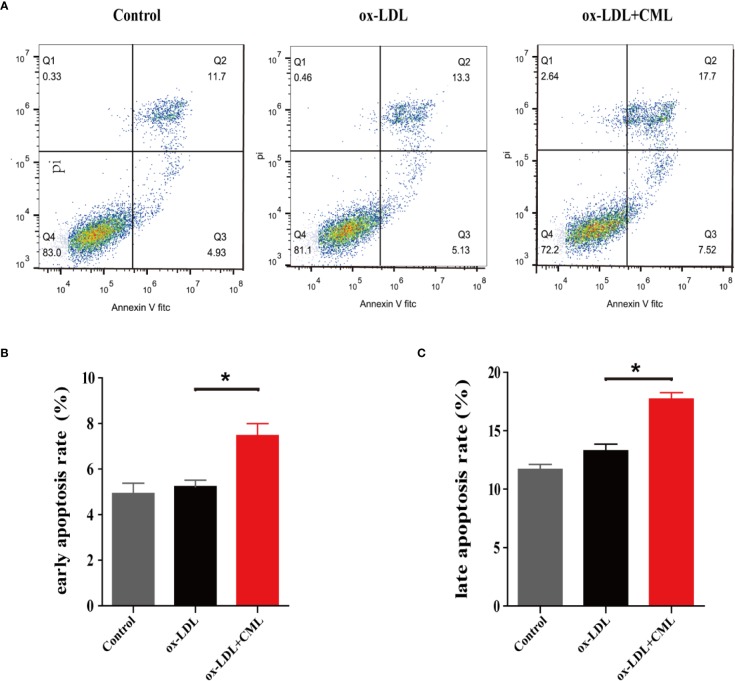
CML induced A7r5 VSMC-derived foam cells apoptosis. A7r5 cells were treated with ox-LDL alone or costimulated with CML for 24 h. **(A)** Apoptosis was detected by Annexin V/PI double-staining flow cytometry, and normal cells, early apoptotic, late apoptotic and necrotic cells in groups of control, ox-LDL, ox-LDL+CML were present in the lower left, lower right, upper right, and upper left quadrants. **(B)** The early apoptosis rate of A7r5 cells apoptosis by flow cytometry. **(C)** The late apoptosis rate of A7r5 cells apoptosis by flow cytometry. Values are expressed as the mean ± SD. *P < 0.05. Annotations: CML, Nϵ-carboxymethyl-lysine; VSMC, vascular smooth muscle cell; ox-LDL, oxidation-low density lipoprotein.

As shown in **Figure 4**, Annexin V/PI double-staining flow cytometry was used to detect cell apoptosis, and normal cells, early apoptotic, late apoptotic, and necrotic cells were present in the lower left, lower right, upper right, and upper left quadrants, respectively (**Figure 4A**). Based on the flow cytometry analysis data, the early apoptosis rate of A7r5 cells gradually increased in the control group, ox-LDL group and ox-LDL+CML group (4.93% ± 0.32% vs. 5.13% ± 0.22% vs. 7.52% ± 0.38%) (**Figure 4B**). Similarly, the late apoptosis rate of A7r5 cells gradually elevated in the control group, ox-LDL group and ox-LDL+CML group (11.70% ± 0.21% vs. 13.30% ± 0.35% vs. 17.70% ± 0.43%) (**Figure 4C**). Significant differences in the early apoptosis rates and late apoptosis rates of A7r5 cells were detected between the ox-LDL+ CML group and ox-LDL group (*P* < 0.05), which manifested that CML exacerbated the apoptosis of A7r5 VSMC-derived foam cells.

### CML Accelerated the Calcification of A7r5 VSMC-Derived Foam Cells

**Figure 5:**
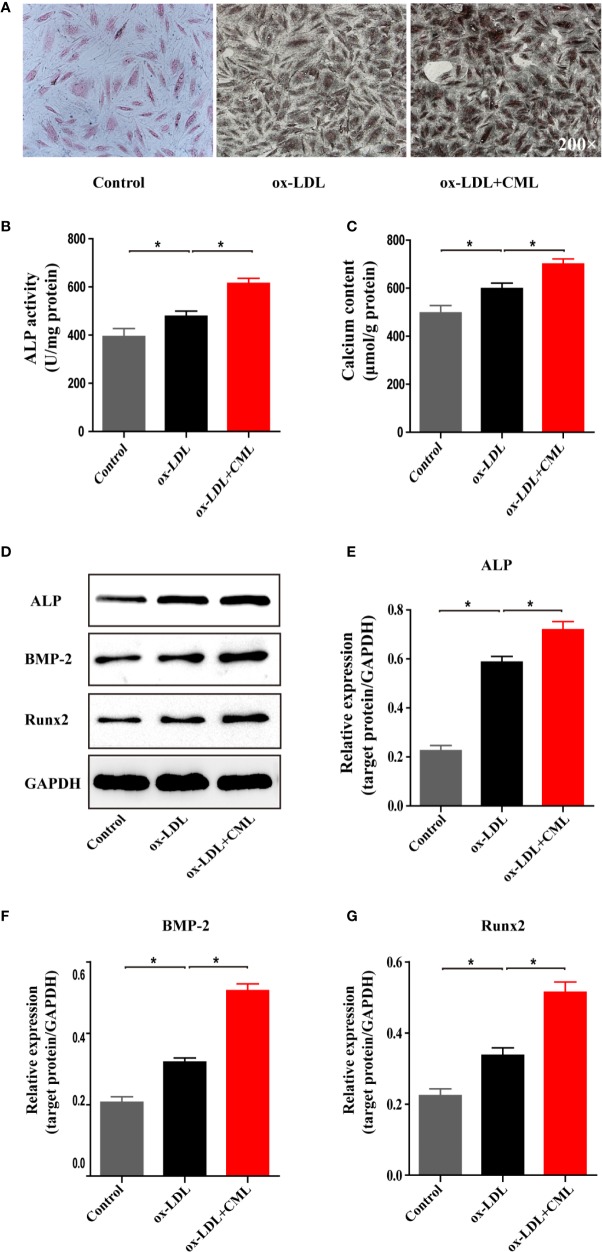
CML promoted VSMC-derived foam cells calcification. **(A)** Representative images of A7r5 cells treated with ox-LDL or ox-LDL and CML by Von Kossa staining (200× magnification). The activity of ALP was analyzed by an ALP activity assay kit. Calcium depositions were measured utilizing the o-cresolphthalein complexone method and normalized in accordance with the cellular protein content. Calcification related proteins (ALP, BMP-2 and Runx2) expression were detected by the western blotting. Quantitative results of **(B)** ALP activity, **(C)** calcium content. **(D)** Western blotting bands of ALP, BMP-2 and Runx2 protein expression. Semiquantitative analysis of western blotting for **(E)** ALP, **(F)** BMP-2 and **(G)** Runx2. Values are expressed as the mean ± SD. *P < 0.05. Annotations: CML, Nϵ-carboxymethyl-lysine; VSMC, vascular smooth muscle cell; ox-LDL, oxidation-low density lipoprotein; ALP, alkaline phosphatase; BMP-2, bone morphogenetic protein 2; Runx-2, runt-related transcription factor 2.

The amount of calcification in A7r5 cells was determined by Von Kossa staining (**Figure 5A**). Compared with the control group, A7r5 treated with ox-LDL exhibited more calcification, and calcification in the ox-LDL+CML group was more serious than that in the ox-LDL group. Similar results were observed for the calcium content and ALP activity. The quantitative analysis results indicated that the ALP activity increased by 21.2% (478.30 ± 9.48 U/mg vs. 394.50±14.54 U/mg) in the ox-LDL group compared with the control group and that ALP activity increased by 28.5% (614.50±9.51 U/mg vs. 478.30±9.48 U/mg) in the ox-LDL+CML group compared with the ox-LDL group (**Figure 5B**). Similarly, the calcium content in the ox-LDL group increased by 20% compared with that in the control group (598.50±10.20 mmol/mg vs. 496.70±13.89 mmol/mg). The calcium content significantly increased by 17% in the ox-LDL+CML group compared with the ox-LDL group (700.50±9.48 mmol/mg vs. 598.50±10.20 mmol/mg) (**Figure 5C**). All the differences found in the comparisons above were statistically significant (*P* < 0.05).

The osteoblastic morphogens BMP-2, Runx2, and ALP are important components of the osteogenic trans-differentiation of VSMCs. We detected the effect of CML on VSMC osteogenic transdifferentiation by assessing the expression of BMP-2, Runx2 and ALP by Western blotting (**Figure 5D**). Compared with the expression levels in the control group, the expression of ALP, BMP-2, and Runx2 in VSMCs were upregulated by ox-LDL treatment by 2.59-fold (0.587±0.016 vs. 0.226±0.0014), 1.54-fold (0.319±0.008 vs. 0.207±0.011), and 1.5-fold (0.3373±0.0149 vs. 0.224±0.013), respectively. Compared with the expression levels in the ox-LDL group, the expression of ALP, BMP-2, and Runx2 in VSMC-derived foam cells was upregulated by CML and ox-LDL costimulation by 1.22-fold (0.7187±0.023 vs. 0.587±0.016), 1.62-fold (0.5193±0.013 vs. 0.319±0.008), and 1.53-fold (0.5145±0.0202 vs. 0.3373±0.0149), respectively (**Figures 5E–G**). All the differences identified in the comparisons above were statistically significant (*P* < 0.05). The results demonstrated that CML aggravated the calcification of A7r5 VSMC-derived foam cells.

## Discussion

DM has increasingly become a global health care problem and causes mortality worldwide due to its complications. VC is highly prevalent in patients with DM, accounting for substantial adverse cardiovascular events (Harper et al., 2016; Chao et al., 2019). DM and VC share several common pathogenic mechanisms and are intrinsically linked (Lanzer et al., 2014; Kay et al., 2016). Long-term hyperglycemias in DM results in the formation and accumulation of AGEs, which accelerates medial calcification and promotes bone matrix protein expression and ALP activity (Laakso and Cederberg, 2012; Zhu et al., 2018; Wei et al., 2019). Inflammation and reactive oxygen species (ROS) production are considered as the pivotal components in the pathogenesis of VC (Chao et al., 2019). Receptors for advanced glycation end production (RAGEs), important receptor for AGEs, have been reported to persist in unstable plaques with microcalcifications by colocalizing with inflammatory cells and VSMCs undergoing osteochondrogenic differentiation (Menini et al., 2013). AGE/RAGE signal have also reported to be the important factor in diabetic VC through mediating oxidative stress in the phenotypic switch of VSMCs *via* the signaling cascades such as TGF-β, NF-κB, and Nox-1. The interaction of AGEs with RAGEs could activate PKC-ζ to trigger downstream signaling through p38 MAPK and NF-κB (Tada et al., 2013; Ott et al., 2014; Kay et al., 2016). Our previous studies have suggested that the CML/RAGE signal mediates microcalcification in diabetic atherosclerotic *via* the p38 MAPK pathway (Wang et al., 2016). However, the underlying mechanism remains poorly elucidated.

VC is similar to orthotopic bone formation, which is involved in the dynamic balance between calcification-inhibiting factors and calcification-promoting factors (Tian et al., 2015; Nakahara et al., 2017). The cells involved in VC include endothelial cells, VSMCs, monocytes and macrophages (Demer and Tintut, 2014). VC is an active process, and VSMCs participate in several associated mechanisms, including apoptosis, osteochondrogenic transdifferentiation, extracellular vesicle release, calcium overload, and cellular senescence (Proudfoot, 2009; Shanahan, 2013; Schurgers et al., 2018; Jaminon et al., 2019). VSMCs can express and release osteochondrogenic proteins *via* osteogenic transformation into phenotypically osteoblast-like cells (Harper et al., 2016; Ma et al., 2017). High phosphate induces a switch toward an osteoblast-like phenotype *via* core-binding factor subunit 1α (Cfba1)/Runx2. Osteogenic-primed VSMCs express ALP and secrete bone-associated proteins such as osteocalcin and osteopontin and bone morphogenetic proteins such as BMP-2 (Jono et al., 2000; Tintut and Demer, 2006).

At present, an increasing number of studies no longer support foam cells as a major hallmark of the early stage of AS. Macrophages have been reported to be the main source of foam cells (Chistiakov et al., 2017), and our previous studies have shown that CML/CD36 accelerated AS progression by promoting the accumulation of macrophage-derived foam cells in the aorta (Xu et al., 2018). Meanwhile, previous studies have reported that VSMC-derived foam cells formation may result in AS calcification and represent a pivotal step in cardiovascular morbidity and mortality. VSMCs can differentiate into macrophages that become foam cells by engulfing lipid (Alexopoulos and Raggi, 2009; Lao et al., 2015). In addition, dedifferentiated VSMCs migrate into the subintimal space of AS plaques to provide structural integrity to the fibrous cap (Samouillan et al., 2012), which also contributes to AS calcification.

In the present study, we built a VSMC-derived foam cells model to investigate the role of CML, a major immunogen of AGEs, in diabetic calcification. Here, we demonstrated that CML promoted diabetic AS induced by VSMC-derived foam cells. First, in CAD patients with DM, the baseline characteristics indicated a poor blood glucose and lipid status. Regarding coronary lesions, more severe coronary lesions were noted in the CAD patients with DM. Then, in an animal study, ApoE^−/−^ mice were used to build a diabetic model *via* STZ injection, which destroyed the *β* cells of the pancreatic islets to promote the formation of endogenous AGEs, followed by administration of a semisynthetic HFD plus injections of CML. After four months, morphological features corresponded to early AS plaques in the ox-LDL group, and the ox-LDL and CML had accelerated AS plaques and cholesterol crystals under the fibrous cap, which is consistent with our previous study (Xu et al., 2018). In addition, ox-LDL and CML can induce extensive spotty calcium deposition and upregulate ALP expression in the aortic AS plaques of ApoE^−/−^ mice. Moreover, we built a foam cell model with VSMCs stimulated by ox-LDL or ox-LDL and CML. CML promoted lipid accumulation in A7r5 derived foam cells as shown by oil red O staining, and VSMC-derived foam cells formation induced apoptosis, which was partly attributed to VSMC calcification (Shi et al., 2017). Previous data showed that inhibition of apoptosis decreased calcification in nodules (Proudfoot et al., 2000). Moreover, apoptotic bodies of dead foam cells and VSMC debris may provide a nucleation microenvironment for calcium hydroxyapatite crystal formation (Johnson et al., 2006). In addition, high glucose can cause endoplasmic reticulum stress-mediated apoptosis, which promotes the development of VSMC calcification (Zhu et al., 2015; Liu and Kong, 2016). Our results suggested that CML augmented the apoptosis of A7r5 VSMC-derived foam cells to aggravate VC of VSMCs.

Referring to the effect of CML on the calcification of A7r5 derived foam cells, we examined the expression of the bone-related proteins BMP-2, Runx2, and ALP. BMP-2 is reported to be a powerful bone morphogenic protein and causes osteogenic transcription (Chen et al., 2017). Runx2 is considered an essential transcription factor for osteogenic gene expression, which is downregulated in normal VSMCs but upregulated in calcified vascular tissue specimens (Evrard et al., 2015). ALP is known to play an essential role in VC and is considered as the key osteoblastic phenotype marker (Sharma et al., 2014). Consistent with the present *in vivo* study, our results revealed that CML increased the calcium content and ALP activity in VSMC-derived foam cells. Western blotting analysis showed that CML upregulated the expression of ALP, BMP-2, and Runx2 in VSMC-derived foam cells to exacerbate VC of VSMCs. These data indicate that CML promotes VSMC-derived foam cells calcification induced by β-GP.

## Conclusion

We concluded that CML promoted VSMC-derived foam cells calcification to aggravate VC in diabetic AS, providing evidence for the contribution of VSMC-derived foam cells to diabetic VC.

## Data Availability Statement

All datasets generated for this study are included in the article/supplementary files.

## Ethics Statement

The clinical study was approved by the Ethical Committee of the First Affiliated Hospital of Xi’an Medical University (Xi’an, China) and carried out in accordance with the institutional guidelines. Written informed consents were obtained from all patients of the First Affiliated Hospital of Xi’an Medical University. The animal study was approved by the Ethical Committee of Affiliated Hospital of Jiangsu University (Jiangsu, China) and conducted in accordance with the Guidelines for Animal Experimentation of the National Institutes of Health.

## Author Contributions

All authors listed have contributions to this work. S-NX and XZ are responsible for performing the experiments, analyzing the data and writing the manuscript. C-JZ, K-LZ and C-XL focus on collecting and analyzing clinical data in human study. WQ, JZ, H-JL, LX, ZS, and K-LZ participate in the animal study and cell study. H-LC, Z-QW, and A-JZ devote themselves to designing the study, revising and polishing the manuscript. All authors have read and approved the final manuscript.

## Funding

This work was supported by the National Natural Science Foundation of China (No. U1932130, 81770450, 81670229, 8157020, 81500272 and 31700699); the Key Program of Shaanxi Provincial Science and Technology Department (No.2017ZDXM-SF-029); the Key Program of Shaanxi Provincial Education Department (No.18JS102); the Talents Program of Xi’an Medical University (No.2015RCYJ01); the Science and Technology Development Research Project of Shaanxi Province-Key Problem of Science and Technology in Social Development (No. 2016SF-034), and the Open Program of Shaanxi Key Laboratory of Ischemic Cardiovascular Disease (2018ZDKF05).

## Conflict of Interest

The authors declare that the research was conducted in the absence of any commercial or financial relationships that could be construed as a potential conflict of interest.
